# Therapeutic plasma exchange in the intensive care unit and with the critically ill, a focus on clinical nursing considerations

**DOI:** 10.1002/jca.21984

**Published:** 2022-04-06

**Authors:** Ian Baldwin, Sarah Todd

**Affiliations:** ^1^ Department of Intensive Care Austin Hospital Melbourne Victoria

**Keywords:** AKI, critical illness, ICU, nursing, TPE, TPE management

## Abstract

Therapeutic plasma exchange (TPE) is a blood purification technique removing antibodies and plasma proteins to modulate disease and promote recovery. The procedure has different methods, using a membrane or plasma separator with many elements similar to continuous renal replacement therapy (CCRT) in the Intensive Care Unit (ICU). These nursing knowledge and skill sets apply where ICU nurses are providing TPE with increasing need. However, different care models are also in place where TPE is the responsibility of apheresis and nephrology teams visiting the ICU. The plasma replacement volume and prescribing is aligned with published guidelines but is variable when critical illness overlays the primary indication for TPE. There are some important considerations for TPE with respect to anticoagulation, machine settings, prescribing, and associated nursing management. TPE can be performed concurrent with CRRT in acute situations using Y‐piece and valve connectors and is a new and recent advanced blood purification for the ICU.

## 1 INTRODUCTION AND BACKGROUND

Plasma exchange is a therapeutic intervention for antibody removal causing disease or toxic states^1^, ^2^ and may be performed in the ICU when acuity mandates admission. Historically, Guillain Bare syndrome (GBS) has been the more common indication when ventilation failure is apparent and ICU care necessary.^3^, ^4^, ^5^ TPE is possibly becoming more frequent in the adult ICU,^6^ and used to modulate the immune system with many organ failure states.^7^, ^8^ Most recently the inflammatory state and organ failures associated with COVID‐19 has been treated with TPE.^9^, ^10^, ^11^ The procedure can be with different extracorporeal (EC) techniques, and when via a membrane or plasma separator has many similarities to the use of convective mode hemofiltration,^12^ that is, plasma removal and replacement but using a substitute such as albumin or thawed Fresh Frozen Plasma (FFP). The procedure is not without side effects and hazards,^13^ and can associate with the volume, and type of plasma substitute.^14^ For example, when large volume FFP administration is used citrate dosage can reduce the ionised calcium and magnesium levels, and TPE does affect the clearance of therapeutic drugs.^14^, ^15^

This article aims to describe use of TPE in the ICU, procedural elements for this to guide and instruct nurses, give examples of the prescribing needed with a paper or e‐document, highlight safety and quality checks, discuss scheduling and nursing planning required for the most efficient therapy in association with a critically ill patient. In addition, where acute kidney injury (AKI) may also be present there is a need to switch the focus each day and timing for best use of each intervention can be difficult. However, this aspect of TPE is advancing where both CRRT and TPE can be done concurrently^16^, ^17^, ^18^ as an advanced procedure and we also describe here.

## 2 NURSES AND TPE

Nurses in the ICU can provide this using knowledge and skills from CRRT, however not all ICU's do this. This can be due to different prescribing and disease management pathways such as hematology and nephrology. In this context, the daily procedure may be done by a nurse or technician external to the ICU.^19^ However, as experienced with dialysis and AKI support for patients in ICU, it is becoming necessary for the ICU to prescribe and perform TPE independently, using their CRRT expertise but for a different and new therapy.

Our ICU nurses provide TPE, however this is a variable situation possibly associated with bed size and other demographics. Smaller centers may provide TPE because they have patients with ventilator dependency such as GB syndrome in their care, and no local apheresis or nephrology nurses to provide TPE. Larger centers, where more complex surgical and supportive interventions are done, and are specialist referral centers may provide TPE frequently, and provided by specialist groups external to ICU available at large facilities. Policies are variable and the procedure is also different amongst the ICU's where TPE is done, for example, blood flow rates, fluids used, time allocated for a treatment. However, CRRT machines with the option for TPE are being used in most cases where ICU nurses are the operators. In larger ICU's and where ICU nurses are not involved, alternative apheresis devices are more likely to be used, for example, centrifugal machines and not with a membrane and associated EC.

## 3 WHAT IS TPE OR PLASMA EXCHANGE?

**FIGURE 1:**
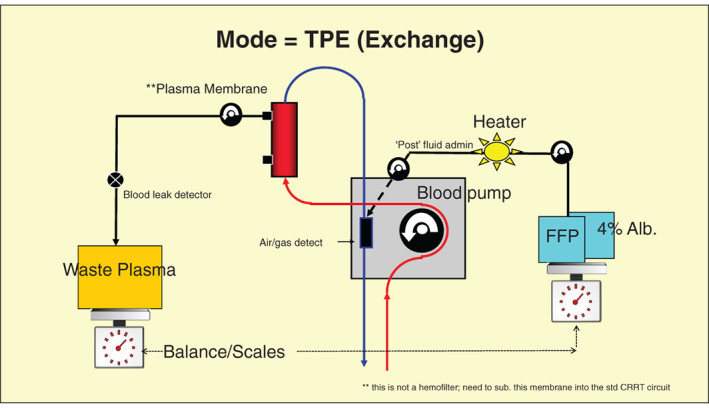
TPE circuit diagram indicating key elements associated with CRRT machines

The name or title for the procedure is essentially the same and is used for a procedure conceptually simple. The membrane or plasma separator placed into the EC allows the passage through, and across the membrane fibers large protein sized molecules (~ 70‐3 000 000 Da) inclusive of plasma water; plasma removal and loss.^7^, ^20^, ^21^ Importantly, this waste plasma inclusive of other proteins and antibodies, drugs and coagulation factors must be replaced^12^, ^22^, ^23^ and not administered prior to the plasma membrane where it would be subject to immediate removal. There are options, but in order to prevent this and maintain blood osmolarity and oncotic pressure, FFP or Albumin solution (4‐5% Albumin) is best administered into the EC after the membrane (to the venous return) as “postmembrane dilution” as done with CRRT^1^, ^22^, ^23^, ^24^ and is conceptually illustrated in Figure 1. This is best to accurately control plasma replacement with a pump device and to access the safety of the bubble trap and air detection sensor on a TPE or CRRT machine. The volume exchanged or rate per hour to provide a total plasma volume between 2 and 4 L over 3 or 4 h may be ~1 L/h. Plasma replacement as removed (an exact exchange) is vital and to be controlled, as hypovolemic arrest would occur quickly if plasma is removed and not replaced.^1^, ^6^, ^19^, ^24^ Fluid removal or negative fluid balance is not appropriate with this procedure.^24^

## 4 UNDERSTANDING THE EXCHANGE VOLUME OR RECIPE

The volume of plasma replacement links to the patient size and BMI with an approximate “one plasma volume” estimation in adults being 50 ml/kg.^5^, ^25^ Another calculation equation is 0.07 × (weight in kg) × (1‐HcT).^12^ For an approximate 60% reduction in plasma macromolecules 1.4 times this plasma volume is considered necessary, for example, for a 70 kg patient with HcT = 55 the plasma volume is 2.69 L and for the 60% reduction in macromolecules or 1.4 times this volume equals 3.7 L in total. This volume exchanged may be rounded up to 4 L where FFP or Albumin is used and provided in 500 ml bottles or bags and the supplied volume may be greater than calculated volume, that is, increased to not waste this fluid. Body weight and other determinates in the calculation may contribute to an under dosing, therefore an increase or higher volume to “use up” or empty any FFP or Albumin is sensible and reasonable. It is also important to remember the machine circuit, with fluids pathways are dead‐space volume and slightly higher volume settings provide for this, and create a need to flush tubing EC dead‐space at the end of a treatment.

The procedure for fluids used,^26^ time frame or duration is not fixed or associated with any supportive data or best practice, but 2 to 6 h is a range we identified in the literature with an understanding plasma removal and replacement must be done carefully.^25^ The drag or movement of plasma across the membrane should be gentle and slow (~1‐1.5 L/h) and with the substitution into the venous return limb of the EC and system some recirculation and mixture does occur and suggests a slower process should be allowed to minimize this and useful for antibody and protein 'activity' or interaction prior to removal,^20^, ^24^ that is, a longer time (~4 h) may be best.

TPE membranes are fragile, and with higher demand (negative pressure drag) and transmembrane pressure, fibers may start to leak blood or other cells. This also means a time period for 4 L over 3 or 4 h works well. Longer times may be done, however this can also be time off CRRT in some cases with concurrent AKI and no fluid balance control being provided. In addition, the circuit can clot and fail with longer duration associated, despite anticoagulation.^27^

## 5 ANTICOAGULATION

The TPE circuit may clot or clog within the membrane or bubble trap and similar to the experience with CRRT. Anticoagulation and the need for this should also be based on an assessment of the patient and their clinical situation and bleeding risk.^14^, ^28^ In some TPE indications such as Thrombocytopenia Purpura (TTP),^12^ the platelet count may be very low^27^, ^29^ and <50 K, no anticoagulation is safest and with a max. four hours treatment time, the circuit will usually function without any clotting and no anticoagulation can be appropriate.^28^ In some cases, the first treatment or exchange procedure does fail or clog within 1 h (eg, hyperlipidemia) and this reflects blood laden with foreign and active and reactive cells and complex pathways with clotting in the EC environment. In the next or subsequent treatments this may subside. Heparin, Epoprostenol, or combining these together, and other heparinoids can be all effective for the task,^14^, ^28^ but higher dosing is necessary where these agents will be cleared or removed in the waste plasma.^14^ This reduces patient exposure, but without higher dosing (×1.5 normal dosing) they may not be effective. It is important to remember the exchange may provide many clotting factors and proteins when FFP is used, and do activate heparinoids via the antithrombin‐׀׀׀ (AT‐3) pathway,^15^ but this also provides sodium citrate in the FFP from the source as a whole blood donation pack. Citrate will reduce the ionised calcium^14^, ^21^ and this is a useful contributor to the function of the EC, but not when 4% to 5% Albumin is used alone.

## 6 TPE AND iCa

The use of FFP for TPE is also administering a dose of sodium citrate (used for the initial whole blood donation), and this will lower the ionised calcium (iCa) level as measured by an arterial blood sample.^21^ Albumin 4% or 5% and or thawed Fresh Frozen Plasma (FFP) is used as replacement fluid for TPE and up to 4.5 L in adults. FFP contains sodium citrate at donation to chelate or lock blood calcium and prevent clotting by lowering serum ionised or free calcium (iCa, *normal range*, 1.13‐1.3 mmol/L). It is estimated 40 g/L or 136.1 mmol/L is included for each three bags of supplied FFP (personal communication with Austin Health Hematology Dept. August, 2021), or 204.12 mmol of sodium citrate for a 4.5 L plasma exchange. Some patients, less FFP is used however calcium levels during TPE are not well understood and this ion may need supplementation with use of TPE and more with 100% FFP use.

We referred to a quality audit (Austin Health Research and Quality week, October 2021, abstract only) of 10 patients from our ICU over the period of 2019 to 2021 that received TPE. The purpose of this audit was to gather data on the iCa levels prior, during and post the treatment as we hypothesized a larger decrease in levels would occur in treatments that used large doses of FFP and associate with the high sodium citrate dose.

This audit allowed us to gather secondary data such as the indication for TPE, the combination of prescribed replacement product, and the number of treatments each of these patients received, which from this group ranged from 1 to −14.

This yielded 50 TPE's in 10 patients with median age of 51 years, 77 kg and 4 h and 4 L exchange and with FFP or mixtures of FFP and Albumin (*n* = 31) fluid and 19 with Albumin alone. Median iCa for the cohort for pre, during and after TPE was 1.20, 1.04, and 1.10 with a minimum case at 0.84 mmol/L during both mixture and 100% Albumin fluid TPE. For the Albumin group, median iCa was 1.20, 1.14, and 1.13. Therefore, iCa does reduce with the use of FFP during TPE and a greater reduction with FFP alone as the replacement plasma; a citrate bolus. In some cases calcium should be given (eg, Calcium Chloride 20 mmol IV or oral dose) as a premed or during the TPE, with a check for the iCa level during the procedure being a routine and sensible policy inclusion.

## 7 MACHINES AND SETTINGS FOR TPE

TPE is an option on most conventional CRRT machines,^30^ with a TPE membrane in the usual circuit kit‐set. In our ICU we use both the Infomed HF 440 (Infomed, Geneva, Switzerland) and Gambro Prismaflex machines (Baxter‐Gambro Lyon, France). Machines are primed and prepared with the TPE mode selection, or similar option, and primed with crystalloid fluid using similar programming to convective clearance mode or hemofiltration,^30^, ^31^ that is, not diffusive mode hemodiafiltration. On completion of priming the machine will offer settings to indicate the total volume for removal and replacement (exchanged) and a time frame for this is achieved by setting the hourly rate of exchange. Another machine prescription is to set the percentage of blood flow rate for plasma removal, for example, a blood flow rate 120 ml/min and an exchange at 10% will be a plasma removal and replacement at 12 ml/min or 720 ml/h. If the total volume for exchange is 3.6 L, this would be 5 h duration (eg, 3600/720 = 5). Alarms for TPE are using the same panel and display and are no different for the EC blood flow and monitoring. The transmembrane pressure will be lower with a TPE membrane and with the fluid removal rate, and require a much lower alarm setting, for example, < 60 mmHg.^22^, ^32^ Blood leak detection can be problematic as the plasma removed is dark and sometimes with a crimson or red tinge making the sensor respond to “blood detection in waste fluid” or similar. This alarm can be re‐calibrated in some machines during the TPE, however, the combined alarms for excessively “high TMP” and “blood leak detection” are likely to be reflective of hemolysis occurring where the alarm should not be simply re‐calibrated and the procedure terminated. A routinely used urine analysis strip test for blood from the waste plasma fluid may be useful.

## 8 TPE: THE PROCEDURE

TPE requires a treatment order to be prescribed by the treating ICU consultant based on the patients clinical indication, weighted dosing calculation and preferred fluid replacement.^32^ This should be carefully and safely considered and prescribed using a dedicated treatment form with pre‐set defaults and local guideline association. In our ICU we have a prescription form to assist with correct prescription and to optimize safety during the setup and running of treatment by the ICU nurse (see Prescribing form example in Appendix S1). Once the correct circuit and membrane are primed with a crystalloid solution (eg, Hartmann's solution) the ICU nurse is able to add the prescribed replacement fluids where, as previously stated, the patient receives post dilution via the venous return line of the EC (after the TPE membrane in the EC).

The machine setup prior to commencing TPE requires a set blood flow, total plasma volume and plasma‐blood % setting. Due to the fragile TPE membrane a blood flow 120 to 150 ml/min is suggested to deliver the treatment effectively whilst ensuring the flow is fast enough to reduce clotting in the membrane. The total plasma volume should be calculated to include an extra max. 200 ml of crystalloid flush at the end of treatment to ensure all Albumin or FFP is delivered to the patient inclusive of dead space in the circuit or fluids heater cassette or other tubing. The plasma‐blood percentage method is recommended to start TPE at 10% with the ability to titrate the percentage by ideally no more than 15% maximum to achieve the targeted treatment time.^33^ TPE is a procedure with a short duration that performs removal and replacement with no option for fluid loss and is not used to perform ultrafiltration and fluid loss.

## 9 PLANNING AND SCHEDULING: NURSING CONSIDERATIONS

TPE in ICU is a complex and advanced procedure that requires thoughtful discussion, planning and team work for successful scheduling and timing.^34^, ^35^ This approach also includes nurse allocation, awareness of best delivery time in respect of drugs given on the day, electrolyte and acid base stability and hemodynamic monitoring and support. These key considerations are listed and summarized in Table 1. Checklists and prescribing documents are very useful and provide safety, quality, and confirm learning.

**TABLE 1:** ICU, apheresis or nephrology nurses providing TPE: planning and clinical considerations associated with TPE in ICU

Timing of commencement	We recommend the treatment be delivered during day time hours with the preferred avoidance of shift change overlap where possible
Nursing allocation	TPE requires an experienced critical care nurse with advanced understanding of the fundamentals of CRRT and the management of an EC for potential troubleshooting
Drug removal	Due to removal of larger molecules and proteins, drugs are cleared readily. This may require administration of these drugs post completion of TPE, for example, rituximab is a key agent often used to suppress antibodies^15^, ^36^
Electrolyte management	The monitoring and potential need for electrolyte replacement such as Ca, K+, and MgSO_4_ ^18^
Hemodynamic instability	As with any EC therapy, hemodynamic^36^ instability due to fluid shifts and exposure to foreign plasma replacement fluids may require vasopressor support as required^1^
Acid base disturbances	Critically ill patients who also require CRRT for an AKI will lose this treatment time when TPE is implemented. This needs to be considered in daily care planning and promotes the concurrent use of TPE and CRRT^18^ from one vascular access (see below).

## 10 FLUIDS USED WITH TPE: ALBUMIN VS FFP

Apheresis guidelines^5^ make recommendation for the use of TPE fluid prescription, that is, FFP, Albumin and other. Most indications for TPE where antibody removal is the goal, substitution for plasma is recommended with 4% albumin. FFP is only recommended for a few indications and TTP is the most frequent for FFP use,^3^, ^5^, ^14^ but practice variation is evident in the literature and within patient demographics and age.^37^ However, where clotting factor depletion is a concern after multiple treatments, a mixture of FFP and Albumin is used. If the prescription requires a mixed percentage, we recommend using 4% albumin replacement fluid separate to the FFP and prior to any FFP use with each procedure. This is without any reference to guideline or evidence, but it does seem logical to remove antibody laden plasma without any FFP in return while the treatment is new and without any EC clotting. FFP as first fluid replacement may also promote clotting with the provision of many factors and including AT‐3, and promote clotting if heparin is used.^38^

Premature clotting with TPE is a failed procedure, delays other interventions, adds to time off CRRT (with associated acid base and biochemistry stability) when AKI is also present, is frustrating for the nurse managing this and can create nursing allocation mismatch or need for over‐time work, and usually a complete re‐start with the repeated nursing work re‐priming and consumables cost(s).

## 11 CONCURRENT CRRT AND TPE

**FIGURE 2:**
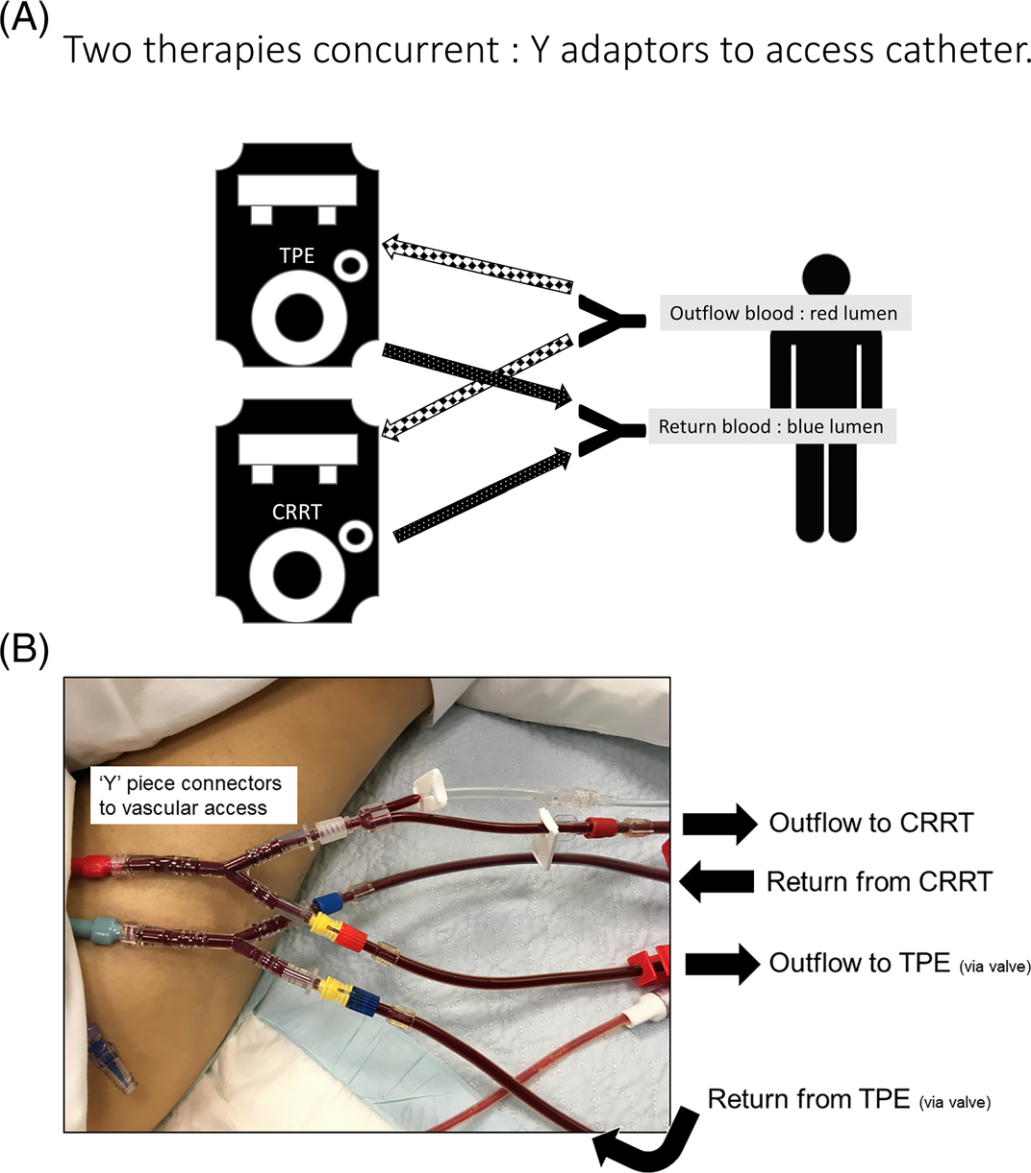
(A) 'Y' piece connectors for two circuits and machines. TPE and CRRT concurrent. (B) Pictured with valves in place

When AKI co‐exists with a need for TPE, and patient acuity raises concern for the time without fluid balance and acid base control (eg, acute hepatic failure and or transplanted organ rejection) CRRT and TPE can be done concurrently, in parallel (see Figure 2A), and has been described since year 2000^39^ with more detailed method in two case studies recently by Kaushik et al.^18^ Pediatric experience for concurrent therapies is reported using centrifugal TPE in “tandem parallel” to CRRT^17^, ^39^ or in series with CRRT and together in parallel to extra corporeal membrane oxygenation (ECMO),^40^ or as a side flow^41^ or reverse parallel with CRRT.^16^ Details for method can be limited in these reports, but three‐way taps into an ECMO or CRRT circuit are mentioned. Our method uses 'Y' piece adaptors connected direct to the vascular access catheter outflow and return lumens. Blood flow is then split to two machines and their EC for both CRRT continuing and the TPE joined in. The join at the split towards the TPE is best done with a dialysis valve adaptor to allow the TPE to be simply connected and disconnected using this valve and luer join. When TPE is complete, the pieces and valves stay in place until the next TPE, and usually the following day. The 'Y' piece on the return line is best connected to a controlled infusion of low‐rate crystalloid or similar to prevent any clotting at the piece. The opposing piece on the outflow is under negative pressure, and clot development does not appear to occur, that is, the valve is adequate alone. Refer to the picture as Figure 2B.

When the TPE is in progress, a shared blood flow to the access is in place, such that the CRRT may require a lowering of usual blood flow, for example, TPE at 120 ml/min and CRRT when usually 200 ml/min is reduced to 120 ml/min also. In some cases, this may require a reduction in CRRT dosing for the period and higher volume CRRT is not possible for this time. However, overall AKI support can be adequate and fluid removal can continue via the CRRT.

## 12 CONCLUSION(S)

ICU nurses are providing TPE for established indications but new and more complex scenarios are emerging as reflected in the COVID 19 pandemic. Antibody, drugs or other proteins and toxins may be suppressed with TPE and the use in any ICU will always be variable and sporadic, and may be provided by either ICU nurses independently or in partnership with apheresis or nephrology nursing specialists. Guidelines are published for TPE and reflect a prescription for use of FFP and or Albumin 4% and 5% solution in a procedure commonly done over 2 to 4 h duration and on repeated days or alternate until an effect is achieved. The procedure can be done with two different approaches and EC but when done by ICU nurses, CRRT machines and a circuit similar to CVVH mode is applied. The key difference is for fluids administration as “post dilution” or after the plasma exchange membrane in the EC. Anticoagulation is also similar to CRRT approaches but when heparin is used, the dose needs be increased due to higher loss with the TPE. All drugs in use will also be removed at a higher rate compared with CRRT. When FFP is used and repeatedly daily, iCa may fall and need supplementation. TPE in association with AKI management and use of CRRT in‐between, requires teamwork and planning to be most efficient. A new method may be used to provide these two therapies concurrently using Y‐piece adaptors with valves on one limb onto the vascular access and when done is an advanced blood purification technique with new learning for advanced practice in the ICU.

## CONFLICT OF INTEREST

The authors declare no conflicts of interest.

## Supporting information


**APPENDIX S1**: Supporting information

## Data Availability

Data sharing not applicable ‐ no new data generated, or the article describes entirely theoretical research
