# Supplementary material for: Therapeutic plasma exchange in the intensive care unit and with the critically ill, a focus on clinical nursing considerations
Source: J Clin Apher. 2022 Apr 6;37(4):397–404. doi: 10.1002/jca.21984 (PMC9539889; doi:10.1002/jca.21984)

Austin HEALTH<br><br>Daily Medical Prescription Chart<br>Therapeutic Plasma Exchange<br>**(TPE)**	U.R Number …………………………………………..<br>Surname ……………………………………………<br>Given Name(s) ……………………………………..<br>Date of Birth ..…………………………………..……<br>**AFFIX PATIENT LABEL HERE**

INTENSIVE CARE UNIT USE ONLY
(Generic : any machine use)

Start Date:............................ Time............................

**Patient weight**<br>_______ **KG**	**TPE PRESCIPTION**<br>**(CHART CAN BE USED FOR THREE DAYS before new replacement)**			
	**Date** →	**Day 1**	**Day 2**	**Day 3**
Mode	**TPE**			
Anticoagulant<br>(see over page for dosing)	**Nil** ☐<br>**Heparin** ☐<br>**Epoprostenol** ☐			
Blood Flow Rate	☐ **120 mL/min.**<br>☐ 150 mL/min.			
**Plasma Exchange Total (A)**	☐ **3.2* L**<br>☐ 4.2* L<br>☐ Other ………….<br>**(*200 ml flush to end)**			
Plasma / Blood ratio (%)**<br><br>*Hrly rate / total volume**<br><br>**(Machine settings vary)**	***Start at 10%**<br><br>****Set hrly rate / total Volume**			
**Fluid (B)**<br>**(Post dilution site)**<br><br>NO fluid loss or removal<br>**A = B**	☐ **Albumin 4%** **…………. mL.**<br><br>☐ **FFP** **…………. mL**			
**Dr Signature & Name**				

DAILY MEDICAL PRESCRIPTION CHART: THERAPEUTIC PLASMA EXCHANGE

Mxx.xx

**Austin HEALTH**<br>Daily Medical Prescription Chart<br>Therapeutic Plasma Exchange<br>**(TPE)**	U.R Number ………………………………………….<br>Surname ……………………………………………<br>Given Name(s) ……………………………………..<br>Date of Birth ..……………………………..…….<br>**AFFIX PATIENT LABEL HERE**

INTENSIVE CARE UNIT USE ONLY

Start Date:.......................... Time..........................

1) Catheter Lock	☐ Heparin	☐ 0.9% Sodium Chloride	
2) Access Type	☐ Bard Niagara Trialysis **(Default)**	☐ Medcomp Trio C-T	
	☐ Other (Permacath etc) ______________________________		

**Anticoagulation**<br>**(refer to Master CRRT policy for more guidance)**	• Higher doses of Heparin may be needed due plasma removal during TPE.<br><br>Heparin: bolus ____________________ (Units)<br>Infusion______________________ (Units/hr)<br>Epoprostenol: Infusion ______________ (ngs/kg/hr)<br>No Anticoagulation __________

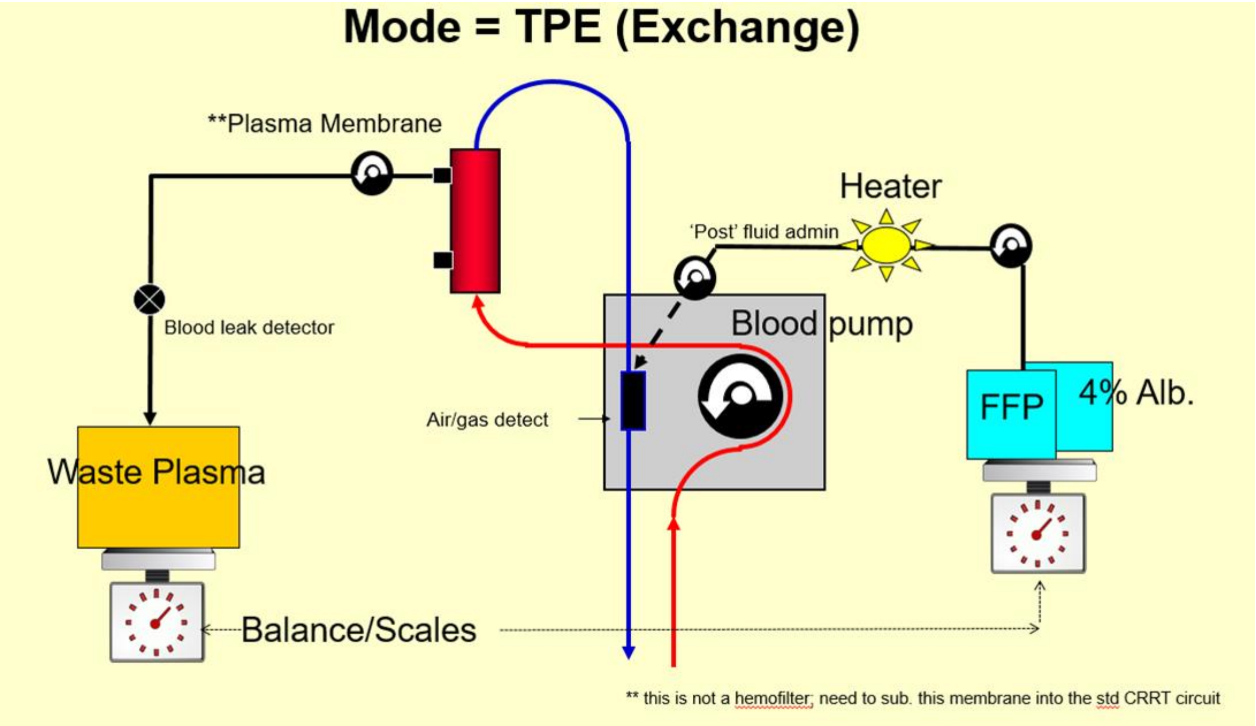

Supplement: Supplementary file 1 — APPENDIX S1: Supporting information [file JCA-37-397-s001.pdf]
